# Mineral biosignature identification from Raman spectroscopy using machine learning

**DOI:** 10.1093/pnasnexus/pgag215

**Published:** 2026-07-14

**Authors:** Yanzhang Li, Anirudh Prabhu, Bingxu Hou, Michael L Wong, Anhuai Lu, Jieqi Xing, Don Ngo, Bo Xu, Robert M Hazen

**Affiliations:** Earth and Planets Laboratory, Carnegie Institution for Science, Washington, DC 20015, USA; Earth and Planets Laboratory, Carnegie Institution for Science, Washington, DC 20015, USA; School of Earth and Space Sciences, Peking University, Beijing 100871, China; Earth and Planets Laboratory, Carnegie Institution for Science, Washington, DC 20015, USA; School of Earth and Space Sciences, Peking University, Beijing 100871, China; School of Earth and Space Sciences, Peking University, Beijing 100871, China; Earth and Planets Laboratory, Carnegie Institution for Science, Washington, DC 20015, USA; State Key Laboratory of Geological Processes and Mineral Resources, China University of Geosciences, Beijing 100083, China; Earth and Planets Laboratory, Carnegie Institution for Science, Washington, DC 20015, USA

**Keywords:** biosignatures, mineralogy, Raman spectroscopy, astrobiology, biomineralization

## Abstract

Biosignature detection remains a key challenge in astrobiology, yet robust mineral biosignatures remain limited. Raman spectroscopy is increasingly applied in planetary exploration, but its high-dimensional spectral information has not yet been fully exploited for biosignature discrimination using data-driven approaches. Here, we integrate Raman spectroscopy with interpretable machine learning to distinguish biotic from abiotic apatite, a ubiquitous phosphate mineral in terrestrial and extraterrestrial environments. We compile 331 apatite Raman spectra from abiotic and biotic sources and extract 21 band-resolved spectral features. Principal component analysis reveals systematic separation between abiotic and biotic endmembers. A random forest classifier achieves 96.8% accuracy on an independent test set. Robustness is confirmed by multiple validation schemes, including leave-one-source-out cross-validation across 60 independent data sources, indicating that model performance generalizes beyond source- or instrument-specific artifacts. Feature importance identifies two dominant controls: phosphate-band broadening as a structural indicator of disorder and the carbonate-band intensity as a chemical signature of substitution. Density-functional calculations reproduce these features in simulated spectra and indicate that carbonate substitution doubles phosphate-tetrahedral distortion and increases formation energies by two orders of magnitude. Mechanically, higher carbonate contents during biomineral apatite formation reduce crystallinity and broaden Raman bands. We propose that the trained machine-learning model and a two-feature decision map enable the rapid probabilistic discrimination of unknown apatite samples. Our Raman-based machine-learning framework establishes a broadly applicable and mission-relevant strategy for deep-time archives and future planetary missions.

Significance statement Identifying reliable biosignatures that can be detected by flight-ready instruments, such as Raman spectroscopy, remains a central challenge in astrobiology. Apatite is a common phosphate mineral on Earth and other rocky bodies, but distinguishing between biological and nonbiological origins is often difficult. Here, we show that biologically formed apatite can be distinguished from abiotic counterparts using interpretable machine learning applied to Raman spectra. The classification is controlled by the width and height of Raman peaks linked to crystal disorder and carbonate incorporation. These spectral signatures remain consistent across multiple Raman datasets and analytical conditions. By linking statistical classification with crystal-chemical distortion and energetics, this study provides a probabilistic screening framework for apatite biosignatures in planetary exploration and deep-time records.

## Introduction

The search for life and biosignatures beyond Earth is a central objective of astrobiology—an effort complemented by attempts to understand the origin and early evolution of life on Earth (1–4). To date, most proposed biosignatures have focused on organic molecules (5, 6), isotopic fractionation (7), or fossils (8) preserved in sedimentary environments. While these signatures have been invaluable for understanding what life is and how life evolves, their preservation and discrimination in extraterrestrial settings are often limited by degradation and ambiguous abiotic pathways (9–11). In contrast, minerals can retain records of biological activity over much longer geological timescales (12, 13), yet mineral-based biosignatures have received comparatively less attention in mission-oriented life-detection strategies. Developing robust mineralogical indicators of life therefore represents a critical and complementary approach for planetary exploration.

Recent Mars missions increasingly rely on flight-ready analytical instruments capable of in situ characterization, including Raman spectroscopy (14), pyrolysis–gas chromatography–mass spectrometry (py-GC-MS) (15), and X-ray fluorescence (16). To fully exploit these mission-limited yet information-rich tools, it is essential to efficiently extract as much diagnostic value as possible from the data they generate. Among all flight-ready instruments, Raman spectroscopy—already deployed on the “Perseverance” rover and planned for deployment on the “Rosalind Franklin” rover—is particularly attractive because it provides bond-specific information on crystal structure and chemical substitution in a nondestructive manner (17, 18). Importantly, Raman spectra acquired by flight-ready instruments are highly comparable to those obtained under laboratory conditions (14, 19), as instrumental parameters generally exert only minor influence on the resulting spectral features. This consistency enables direct translation of laboratory reference datasets to planetary environments. Nevertheless, Raman spectra of natural geological samples are often complex, with overlapping vibrational modes, heterogeneous backgrounds, and chemistry-dependent band shifts (20), making interpretation based on individual peaks or qualitative inspection inherently challenging.

Among candidate mineral biosignatures, apatite-group minerals are especially promising yet underexplored (12, 20). Apatite [Ca_5_(PO_4_)_3_(F,Cl,OH,CO_3_)] is ubiquitous on Earth, Mars, and other rocky worlds and can incorporate a wide range of chemical substitutions, allowing it to record information about biological activity, fluid chemistry, and environmental conditions (21, 22). Previous studies have shown that biologically mediated apatite commonly contains higher carbonate (${\mathrm{CO}}_{3}^{2-}$) content than purely abiotic geological apatite (23, 24) and that such substitution can influence Raman spectral features, including the emergence of ${\mathrm{CO}}_{3}^{2-}$-related vibrational modes and the broadening of phosphate bands (25–27). However, these features have not been integrated into a quantitative, multivariate framework capable of providing objective and transferable discrimination between biotic and abiotic apatite.

These limitations point to a more fundamental challenge: Raman spectra represent high-dimensional, multivariate signals in which multiple features like band positions, widths, and relative intensities vary simultaneously and often nonlinearly. Discrimination of biotic and abiotic apatite, therefore, cannot reliably depend on single spectral features or simple rule-based thresholds. Instead, robust interpretation requires analytical frameworks capable of capturing coupled variations among multiple spectral parameters and translating them into objective, quantitative decision criteria. In this context, recent studies have demonstrated the potential of integrating py-GC-MS with data-driven approaches for biosignature detection (5, 28), enabling the discrimination of biologically derived organic signatures from complex abiotic backgrounds. Such efforts illustrate how AI can extract subtle, multivariate patterns that are difficult to interpret using traditional qualitative or rule-based methods (29–31). So far, analogous applications of machine learning to mineralogical biosignatures remain limited, leaving their potential for distinguishing biotic and abiotic mineral phases largely underexplored.

Here, we address this gap by developing a machine-learning pipeline tailored to Raman spectroscopy for identifying robust mineralogical indicators of biogenesis. We compile a dataset of 331 Raman spectra of apatite samples spanning abiotic geological, synthetic, and biotic origins and apply a random forest (RF) classifier model to identify the most diagnostic spectral features. In addition to ${\mathrm{CO}}_{3}^{2-}$-related signals, the width of the dominant phosphate band emerges as a particularly sensitive and reliable discriminator. These findings reflect increased structural distortion and reduced crystallinity associated with ${\mathrm{CO}}_{3}^{2-}$ incorporation, which raises formation energies and inhibits the growth of well-ordered apatite crystals. By linking data-driven feature selection with crystal-chemical interpretation, this study provides a quantitative and mechanistically grounded framework for distinguishing biotic from abiotic apatite using flight-ready Raman spectroscopy, with direct relevance to future planetary exploration missions.

## Results

### Raman spectroscopy dataset of apatite

**Figure 1:**
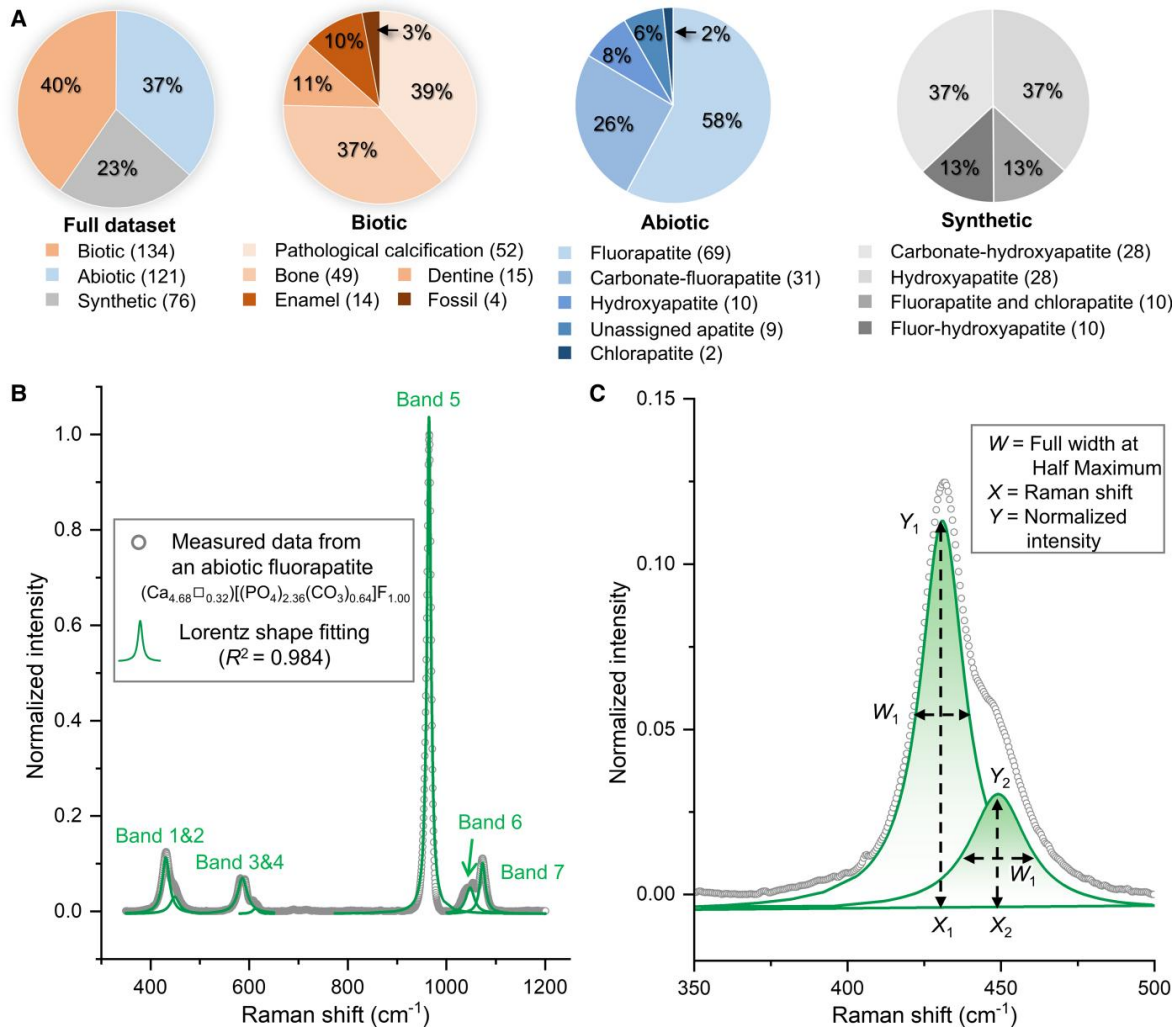
Overview of Raman spectroscopy dataset of apatite. A) The diversity of apatite samples among three classes—biotic, abiotic, and synthetic. B) A natural abiotic carbonated-fluorapatite Raman spectrum with peak fitting using a Lorentz line shape. The spectrum and corresponding chemical formula are from the RRUFF database (https://www.rruff.net/). The symbolic square represents vacancies in the apatite lattice. C) The zoom-in spectra of bands 1 and 2 (from the same *v*_2_ mode of ${\mathrm{PO}}_{4}^{3-}$) and their three peak parameters (full width at half maximum, Raman shift, and normalized intensity) from spectral fitting.

We collated 331 Raman spectra of apatite from publicly available publications, real measurements in the laboratory, and public databases. Most spectra span 300–1,500 cm^−1^ of Raman shift, including spectra measured with different wavelengths (e.g. 532, 614, and 785 nm), but without consideration of polarization effects or crystal orientation. These data thus represent the most easily generated results employing conventional Raman spectroscopy. Among these spectra, only three were collected from different spots on the same grain and four were acquired from the same grain using different excitation wavelengths, and duplicate scans from the same point are excluded. That is, all 331 spectra are strictly from 324 individual apatite grains. We only included apatite specimens with known origin attribute: biogenic, abiogenic, or synthetic (Fig. 1A). The 134 biotic apatite spectra have five subcategories: modern or ancient pathological calcification (52 spectra), bone (49), dentin (15), enamel (14), and fossil (4). The abiotic dataset consists of 121 apatite spectra derived from magmatic rocks and sedimentary phosphorites, including 69 fluorapatite, 31 carbonate-fluorapatite, 10 hydroxyapatite, two chlorapatite, and nine samples without an assigned supergroup name. The third dataset class includes 76 Raman spectra from synthetic apatite with designed chemical anion groups, including carbonate-hydroxyapatite, hydroxyapatite, fluorapatite, chlorapatite, and fluor-hydroxyapatite. Although the discrimination of synthetic apatite is not the target of this study, these data provide a critical component because they can be utilized to simulate those newly formed apatite species in both abiotic and biotic cases. More importantly, synthetic apatite serves as a baseline group that reveals how Raman bands may be altered owing to small particle size and low degree of crystallinity.

In a standard, unpolarized apatite spectrum (Fig. 1B and Table 1), there are four Raman-active internal vibrational modes from the tetrahedral symmetry of phosphate (${\mathrm{PO}}_{4}^{3-}$) group: the symmetric stretching mode (*v*_1_), the symmetric bending mode (*v*_2_), the asymmetric stretching mode (*v*_3_), and the asymmetric bending mode (*v*_4_). Specifically, the *v*_1_ symmetric stretching mode produces the most intense and diagnostic band at ∼960 cm^−1^. The *v*_2_ and *v*_4_ bending modes appear at ∼420–465 and ∼575–620 cm^−1^, respectively, while the *v*_3_ asymmetric stretching mode gives rise to weaker bands in the ∼1,025–1,090 cm^−1^ region. In this study, each mode except *v*_1_ was further divided into two subbands based on those obvious peaks in common apatite spectra, resulting in seven subbands (denoted as bands 1–7 from lower to higher Raman shift, respectively; Fig. 1B and Table 1), e.g. bands 1 and 2 from the same *v*_2_ mode of ${\mathrm{PO}}_{4}^{3-}$. Variations in the positions, intensities, and widths of these bands reflect changes in crystallinity, lattice strain, and chemical substitutions (for example, F^−^, Cl^−^, OH^−^, and ${\mathrm{CO}}_{3}^{2-}$) within the apatite structure. In particular, the substitution of ${\mathrm{CO}}_{3}^{2-}$ gives rise to its symmetric stretching vibration (∼1,060–1,090 cm^−1^, also denoted as *v*_1_) overlapping with the *v*_3_ of ${\mathrm{PO}}_{4}^{3-}$. Generally, band 6 (the one with lower frequency of *v*_3_) has a higher intensity than band 7 (the one with higher frequency of *v*_3_) in ${\mathrm{CO}}_{3}^{2-}$-free apatite, whereas the intensity of band 7 often exceeds band 6 when incorporating ${\mathrm{CO}}_{3}^{2-}$ and its *v*_1_ band (for example, the spectrum of geological carbonate-fluorapatite in Fig. 1B).

**Table 1:** Lines of apatite in Raman spectra.

Number of bands in dataset	Raman shift (cm^−1^)	Fragment	Vibration
1	420–440	${\mathrm{PO}}_{4}^{3-}$	Symmetric bending (*v*_2_)
2	435–465		
3	575–595		Asymmetric bending (*v*_4_)
4	590–620		
5	955–970		Symmetric stretching (*v*_1_)
6	1,025–1,060		Asymmetric stretching (*v*_3_)
7	1,060–1,090		
7	1,060–1,090	${\mathrm{CO}}_{3}^{2-}$	Symmetric stretching (*v*_1_)

Apparently, much of the structural and compositional information is embedded within the characteristics of individual Raman bands rather than in the overall spectral profile. Therefore, spectral deconvolution and peak-fitting procedures are required to resolve overlapping bands and quantitatively extract critical peak parameters, enabling a more complete and physically meaningful interpretation of the Raman data. Prior to peak fitting, all 331 Raman spectra were subjected to baseline correction and intensity normalization, ensuring that variations in baseline offset and absolute signal intensity do not bias subsequent peak fitting and comparative analyses. After conducting the multipeak fitting on the whole normalized spectrum within 350–1,200 cm^−1^ by Lorentz line shapes, full width at half maximum (*W*), Raman shift (*X*), and normalized intensity (*Y*) can be extracted from each band (Fig. 1C). Full-spectrum fitting of 331 Raman spectra yields an average coefficient of determination (*R*^2^) of 0.973, indicating a high fitting quality and validating the accuracy of both the number of fitted peaks and the adopted peak line shape. The fitted peak positions of all spectra are consistently distributed around their standard reference values (Fig. S1). The observed slight deviations are attributed to variations in crystal chemistry among different samples, as well as to instrumental differences between measurements. As a result, the final feature set used for downstream analysis consists of 21 spectral features derived from the seven Raman bands.

### Model performance and critical feature determination

**Figure 2:**
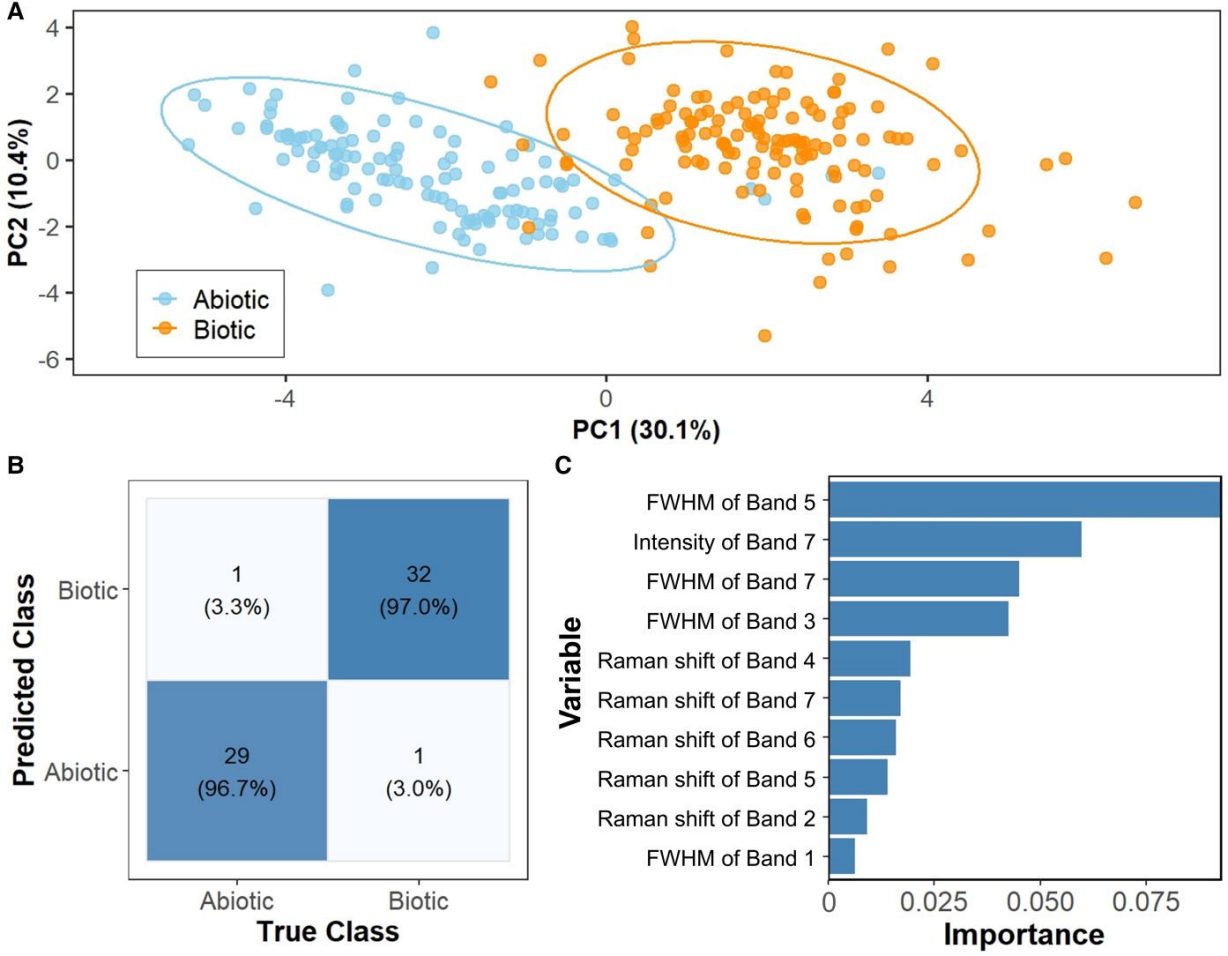
The performance of unsupervised and supervised models on the Raman spectral dataset of abiotic and biotic apatite. A) PCA of abiotic and biotic apatite samples based on 21-dimension Raman-derived spectral features. Scores on the first two principal components (PC1 and PC2) are shown, accounting for a combined 40.5% of the total variance. Points represent individual samples colored by endmember classification, and ellipses indicate the 95% data dispersion for each class assuming a multivariate *t* distribution. B) Confusion matrix for the independent test set obtained from the RF classifier trained on abiotic and biotic endmembers. C) Feature-importance ranking of the top 10 Raman-derived parameters controlling classification performance in the RF model.

We first examined the intrinsic structure of the data using principal component analysis (PCA) based on the spectral feature dataset consisting of 121 abiotic and 134 biotic apatite. The PCA score plot (Fig. 2A) reveals a clear separation between abiotic and biotic apatite along the first principal component (PC1), which explains 30.1% of the total variance, with additional dispersion captured by PC2 (10.4%). PC1 is dominated by spectral widths of band 5 (*v*_1_(${\mathrm{PO}}_{4}^{3-}$)), band 7 (overlapping *v*_3_(${\mathrm{PO}}_{4}^{3-}$) and *v*_1_(${\mathrm{CO}}_{3}^{2-}$)), and band 3 (*v*_4_(${\mathrm{PO}}_{4}^{3-}$)), suggesting structural disorder is the primary source of variation. In contrast, PC2 is mainly influenced by the positions of band 1 (*v*_2_(${\mathrm{PO}}_{4}^{3-}$)), band 3 (*v*_4_(${\mathrm{PO}}_{4}^{3-}$)), and band 4 (*v*_4_(${\mathrm{PO}}_{4}^{3-}$)), associated with variations in local crystal-chemical environments. Although partial overlap exists near the boundary, the two endmembers occupy largely distinct regions in low-dimensional space, indicating that systematic differences in Raman band characteristics are already encoded in the data without imposing any classification constraints. In three-dimensional PCA space (Fig. S2), biogenic and abiogenic apatite remain primarily separated along PC1, whereas projections excluding PC1 show substantial overlap, indicating that the dominant discriminative information is carried by the first principal component.

Building on this unsupervised separation, we trained an RF model for supervised discrimination due to its robustness to correlated variables, ability to model complex feature interactions, and intrinsic feature-importance estimation (28, 32). The dataset with 255 spectra was randomly divided into training (75%) and independent test (25%) subsets, with model hyperparameters optimized using 10-fold cross-validation on the training data (Table S1). The resulting confusion matrix (Fig. 2B) demonstrates robust classification performance on the independent test set, with an overall accuracy of 0.968. Only one biotic apatite is incorrectly predicted as abiotic, and one abiotic sample is misclassified as biotic. Accordingly, the model achieves a sensitivity of 0.970 for biotic apatite and a specificity of 0.967 for abiotic apatite. Consistent with these results, the model attains a high test-set area under the curve (AUC) value of 0.998 (Table 2), indicating excellent discrimination independent of classification threshold. The low and nearly symmetric misclassification rates suggest that the classifier maintains a strict boundary between biotic and abiotic endmembers while avoiding systematic bias toward either class.

**Table 2:** Comparison of accuracy and the AUC value from four cross-validation (CV) operations on training set and the prediction on testing set.

Operations	Accuracy	AUC
Repeated 10-fold CV	0.970	0.998
Leave-one-out CV	0.979	0.997
Leave-one-source-out CV	0.938	0.984
Repeated 10-fold CV under label permutation	0.479	0.481
Prediction	0.968	0.998

We compared model performance across several validation schemes implemented to test robustness and rule out information leakage (Table 2 and Materials and methods). High accuracies (∼0.97) and AUC values (∼0.99) are consistently obtained under repeated 10-fold and leave-one-out cross-validation. More importantly, considering that the 255 spectra collected from 60 distinct sources (54 publications, three public databases, and three sample groups measured on three instruments), the same source could inflate performance if split across training and testing sets. We therefore conducted leave-one-source-out cross-validation, in which all spectra from one source were withheld for testing, to assess whether model performance generalizes across independent sources. This evaluation, achieving a still high accuracy (0.938) and AUC value (0.984), further confirms generalization across independent data sources. In contrast, as a negative control, label permutation was performed by randomly reassigning the abiotic and biotic labels across spectra before model training. In this case, both accuracy and AUC reduced to near-chance levels (∼0.5), consistent with random classification performance. All these results further support that the test-set AUC of 0.998 is driven by real spectral differences rather than overfitting or data leakage. To further evaluate the influence of inter-source analytical variability, we performed additional PCAs comparing internally acquired spectra in our laboratories and public spectra from literature and open-access datasets. Although moderate clustering associated with the analytical source is observed (Fig. S3A), this pattern likely reflects both analytical variability and intrinsic differences among the sampled apatite populations. Nevertheless, separation between biotic and abiotic apatite remains evident across datasets. Importantly, PCA performed exclusively on public spectra still preserves the principal separation trend between biotic and abiotic samples (Fig. S3B), suggesting that the dominant spectral patterns are not solely driven by source-specific analytical effects. Above all, results support the robustness and generalizability of the identified Raman biosignatures across independently acquired datasets and analytical conditions.

**Figure 3:**
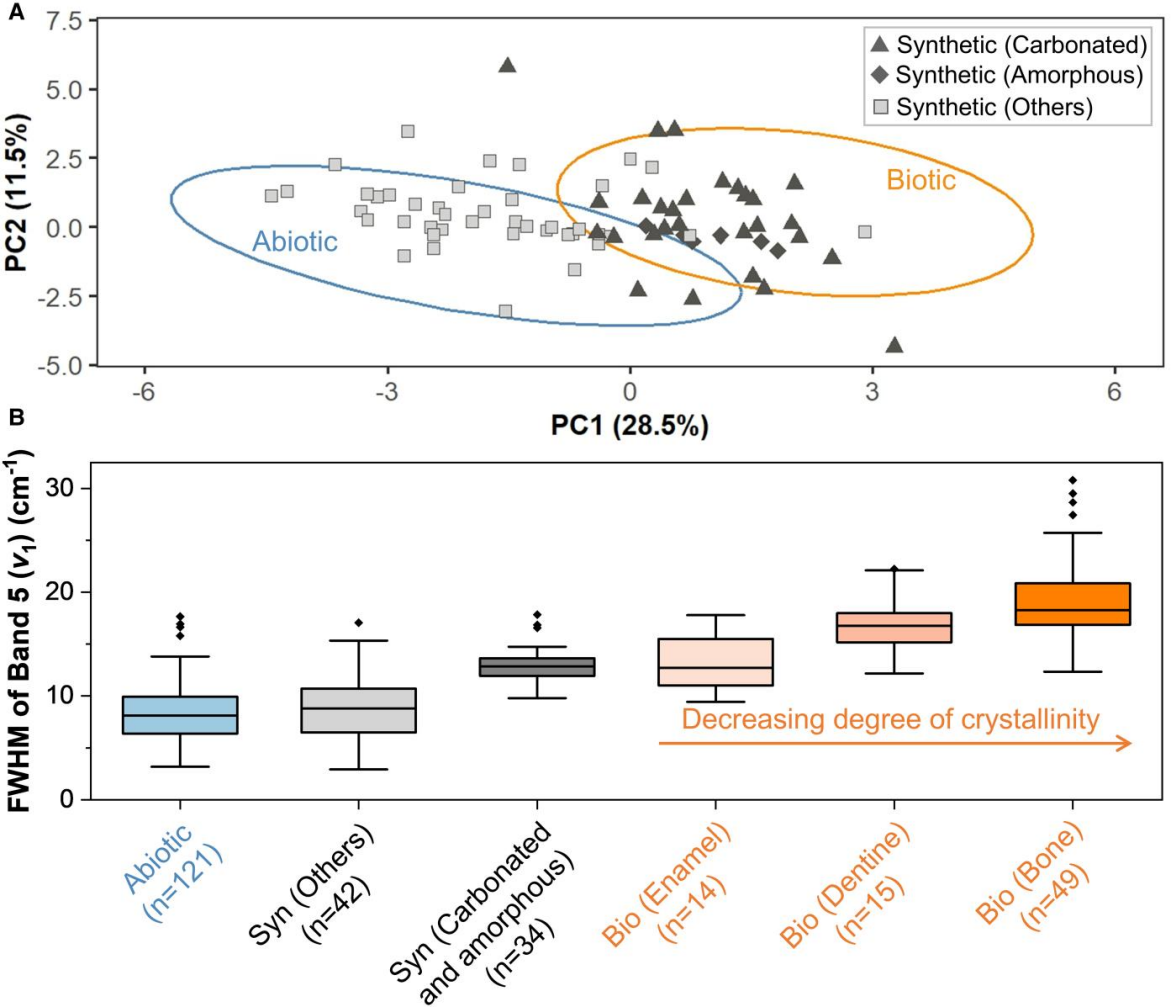
Statistics of different apatite subcategories on representative features. A) PCA of abiotic, biotic, and synthetic apatite samples based on 21-dimension Raman-derived spectral features. Scores on the first two principal components (PC1 and PC2) are shown, accounting for a combined 40.0% of the total variance. For simplicity, the abiotic and biotic data points are hidden, but their ellipses of 95% data dispersion (assuming a multivariate *t* distribution) are shown. Points represent individual synthetic samples from three subcategories. The original PCA figure with all data is shown in Fig. S5. B) Comparison of FWHM of the *ν*_1_ (${\mathrm{PO}}_{4}^{3-}$) band (band 5) among subcategories from synthetic (Syn) and biotic (Bio) apatite. Box-and-whisker plots show the median (central line), 25th–75th percentiles (bounds of the box), and maximum–minimum values (whiskers).

Analysis of feature importance highlights a small subset of spectral parameters that dominate model performance (Fig. 3C). The most influential feature is the full width at half maximum (FWHM) of the *v*_1_(${\mathrm{PO}}_{4}^{3-}$) band (band 5), followed by the intensity and the FWHM of the carbonate-related *v*_1_ band (band 7). Additional important features include the FWHM of band 3, as well as Raman shifts associated with bands 4–7. This result is largely consistent with the main controlling factors identified by PCA. Notably, peak width and intensity parameters consistently rank higher than absolute peak positions, indicating that structural disorder and relative spectral contributions exert stronger control on classification than small variations in Raman shift. These results demonstrate that the RF model identifies physically meaningful spectral features linked to phosphate ordering and carbonate incorporation, rather than relying on spurious correlations.

Using the trained RF model, we calculated the predicted probability of biogenesis for each abiotic and biotic apatite sample. The resulting probability distributions show a clear separation between the two endmembers (Fig. S4). Most abiotic apatite samples exhibit low biogenic probabilities close to zero, whereas biogenic apatite samples cluster at high probabilities approaching unity. Only a small number of samples fall within the intermediate probability range, corresponding to boundary cases near the decision surface identified by the model. This result demonstrates that the classifier provides not only accurate binary discrimination but also a continuous, probabilistic measure of biogenic likelihood for individual apatite samples.

### Abiotic–biotic apatite discrimination factors

To identify the dominant chemical and structural factors that drive the separation between abiotic and biotic apatite in Raman spectral feature space, we further applied PCA to all Raman-derived spectral features from the whole dataset consisting of abiotic, synthetic, and biotic samples (Figs. S5 and 3A). Upon inclusion of synthetic apatite as a third endmember, although the variance explained by PC1 and PC2 changes slightly (from 30.1 and 10.4% to 28.5 and 11.5%, respectively; Fig. 2A), the dominant biotic–abiotic separation is preserved in low-dimensional space, suggesting the robustness of this discrimination. In contrast, synthetic samples show substantial overlap with both abiotic and biotic populations, implying that different synthetic apatite specimens share spectral characteristics with abiotic and/or biotic samples. To explore the structural basis of these spectral signatures, synthetic samples, which were deliberately included in the dataset due to their well-known and well-constrained chemistry and structure, were subdivided and examined in the PCA score plot (Fig. 3A). This analysis reveals substantial overlap between biotic apatite and both amorphous and carbonated synthetic apatite, indicating that these materials share key physicochemical characteristics. Notably, the amorphous apatite samples are freshly prepared ${\mathrm{CO}}_{3}^{2-}$-free fluor-hydroxyapatite, which presented no diffraction peaks in X-ray powder diffractograms (33). Therefore, these patterns indicate that low crystallinity and carbonate substitution are two distinct factors in controlling the discrimination of abiotic and biotic apatite.

Note that the overwhelmingly dominant *ν*_1_ band (i.e. band 5) is most sensitive in response to the chemical and structural alteration of apatite, and this band's FWHM is also the most important among the 21 features in our machine-learning model (Fig. 2C). To quantitatively examine how chemical, structural, and crystallinity-related variation influences the dominant phosphate vibration, we further compare the FWHM of the *ν*_1_ band across six apatite subcategories (Fig. 3B). Abiotic apatite exhibits the narrowest phosphate band, with a mean FWHM of 8.3, followed by synthetic apatite lacking ${\mathrm{CO}}_{3}^{2-}$ substitution or amorphous structure, with a mean value of 8.8. In contrast, synthetic apatite containing ${\mathrm{CO}}_{3}^{2-}$ substitution or exhibiting amorphous character shows a substantially broadened *ν*_1_ band, with a mean FWHM of 13.0. A similar broadening is observed in biotic apatite derived from enamel, which explains to some extent why amorphous and carbonated synthetic apatite can fall into the region of biotic apatite in PCA space (Fig. 3A). As for apatite in dentin, the mean FWHM value is 16.8, while biotic apatite from bone shows the largest band width, with a mean value of 19.4. Notably, apatite in enamel, dentin, and bone is characterized by progressively decreasing crystallinity (22). Therefore, broadening of the FWHM of the *ν*_1_ band is strongly associated with biotic origin.

### Carbonate-altered chemistry, structure, and crystallinity in biotic apatite

Previous studies, largely based on synthetic carbonated apatite, have established empirical linear relationships between *ν*_1_-band FWHM and ${\mathrm{CO}}_{3}^{2-}$ content, and have therefore attributed phosphate-band broadening primarily to increased ${\mathrm{CO}}_{3}^{2-}$ substitution (25–27, 34). However, we suggest that this interpretation is incomplete for two reasons. First, in fully ${\mathrm{CO}}_{3}^{2-}$-free synthetic apatite with an amorphous structure in which long-range order is absent (33), the mean FWHM of the *ν*_1_ band can reach values as high as 12.4, comparable to those observed in biotic apatite. This broadening cannot be explained by ${\mathrm{CO}}_{3}^{2-}$ substitution and instead reflects structural disorder alone. Second, this observation raises a fundamental question as to whether ${\mathrm{CO}}_{3}^{2-}$ incorporation and loss of long-range order are intrinsically coupled, and which of these factors exerts the primary control on *ν*_1_-band broadening.

**Figure 4:**
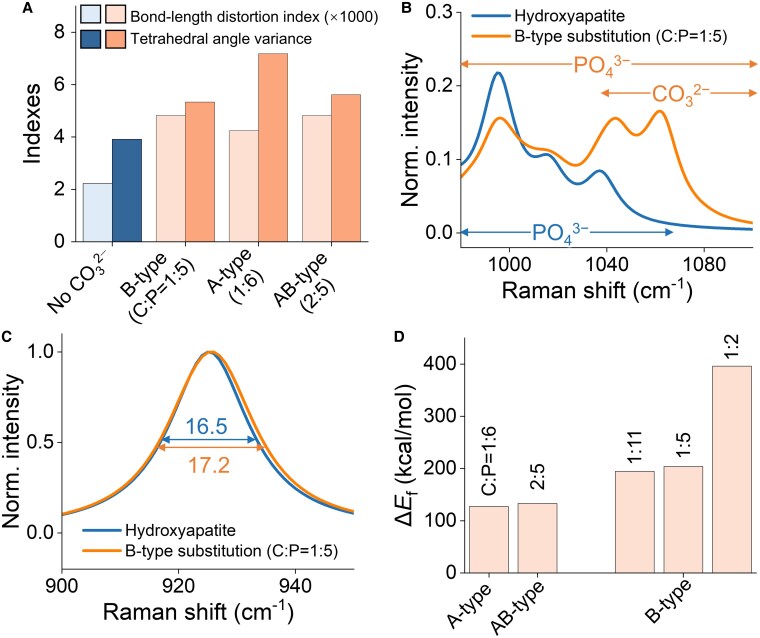
Carbonate-induced structural distortion, Raman band broadening, and formation energetics of apatite. A) Bond-length distortion index and tetrahedral angle variance of ${\mathrm{PO}}_{4}^{3-}$ units in pristine hydroxyapatite and different ${\mathrm{CO}}_{3}^{2-}$-substituted configurations. B) Calculated Raman spectra illustrating the emergence of ${\mathrm{CO}}_{3}^{2-}$-induced bands. C) Calculated Raman spectra illustrating ${\mathrm{CO}}_{3}^{2-}$-induced inhomogeneous broadening of the *ν*_1_ band. For comparison, a slight offset was imposed on the Raman shift to make the two spectra almost overlap. D) Formation energies (Δ*E*_f_) of ${\mathrm{CO}}_{3}^{2-}$-substituted apatite compared with the pristine one. In all panels, the configuration with ${\mathrm{CO}}_{3}^{2-}$ replacing ${\mathrm{PO}}_{4}^{3-}$, OH^−^, and both ${\mathrm{PO}}_{4}^{3-}$ and OH^−^ sites are denoted as B-, A-, and AB-type substitutions, respectively. The C-to-P ratios in different substitution cases are given.

To address these issues, we first quantify local structural distortion induced by ${\mathrm{CO}}_{3}^{2-}$ substitution, evaluating both the bond-length distortion index and the tetrahedral angle variance of ${\mathrm{PO}}_{4}^{3-}$ units (35, 36) (Fig. 4A). In ${\mathrm{CO}}_{3}^{2-}$-free hydroxyapatite, the ${\mathrm{PO}}_{4}^{3-}$ tetrahedron is only weakly distorted, with a bond-length distortion index of 0.0022 and an angle variance of 3.92, consistent with a highly ordered structure. Upon ${\mathrm{CO}}_{3}^{2-}$ incorporation, both metrics increase substantially. For the case of ${\mathrm{CO}}_{3}^{2-}$ replacing ${\mathrm{PO}}_{4}^{3-}$ (i.e. B-type substitution with the C-to-P ratio of 1:5), the average distortion index of the remaining five ${\mathrm{PO}}_{4}^{3-}$ tetrahedrons rises to 0.0048 and the angle variance to 5.33, while A-type substitution (one ${\mathrm{CO}}_{3}^{2-}$ replacing two OH^−^ at the C-to-P ratio of 1:6) yields values of 0.0042 and 7.18, respectively. In the AB-type configuration, where ${\mathrm{CO}}_{3}^{2-}$ substitutes both ${\mathrm{PO}}_{4}^{3-}$ and OH^−^ sites (the C-to-P ratio of 2:5), the distortion index remains high (0.0048) and the angle variance is 5.62. These results show that ${\mathrm{CO}}_{3}^{2-}$ substitution perturbs the local geometry of ${\mathrm{PO}}_{4}^{3-}$ tetrahedrons by increasing both bond-length and angular distortion by approximately a factor of two, which significantly affects P–O stretching and O–P–O bending vibrations, respectively. In addition, carbonate incorporation breaks the equivalence among ${\mathrm{PO}}_{4}^{3-}$ tetrahedrons within the unit cell (Tables S2 and S3), resulting in distinct phosphate groups with different bond lengths and angles. This loss of equivalence leads to the breaking of symmetry in one unit cell and the reduction of long-range structural order.

Raman spectral calculations further provide a mechanistic explanation for the RF results and illustrate how carbonate substitution modifies the vibrational characteristics of apatite at the crystal-chemical level. Compared with the experimental spectra, most simulated spectral features (e.g. band count and relative intensity) can be reproduced (Fig. S6). At the region of band 6 (the lower frequency one of *v*_3_ at 980–1,030 cm^−1^) and band 7 (the one 1,030–1,090 cm^−1^ from the *v*_3_ of ${\mathrm{PO}}_{4}^{3-}$ and possibly the *v*_1_ of ${\mathrm{CO}}_{3}^{2-}$), asymmetric stretching of P–O bonds in ${\mathrm{CO}}_{3}^{2-}$-free hydroxyapatite can bring about the Raman-active vibration at 1,040 cm^−1^ (Fig. 4B). Importantly, it successfully supports the RF results, which identify the intensity and width of band 7 as key diagnostic features of biotic apatite. Using B-type substitution as a representative case (the C-to-P ratio of 1:5), incorporation of ${\mathrm{CO}}_{3}^{2-}$ not only extends ${\mathrm{PO}}_{4}^{3-}$ band to higher frequency (up to 1,090 cm^−1^) but introduces additional vibrational features at 1,036–1,090 cm^−1^. It should be noted that, due to the limitation of computational capacity, the calculated Raman spectra are based on long-range ordered crystalline models and therefore do not explicitly capture the extensive structural disorder present in natural apatite. Nevertheless, even within a single substituted unit cell, ${\mathrm{CO}}_{3}^{2-}$ incorporation breaks the equivalence of ${\mathrm{PO}}_{4}^{3-}$ tetrahedrons, producing multiple local P–O bond lengths and closely spaced *ν*_1_ vibrational modes. When these discrete modes are convoluted, an apparent change of the *ν*_1_ band (i.e. inhomogeneous broadening) emerges, with the effective FWHM increasing from ∼16.5 to ∼17.2 cm^−1^ in our calculations (Fig. 4C). In natural biotic apatite, this effect is expected to be substantially amplified by variable carbonate content, mixed substitution types (A, B, and AB types), and diverse charge-compensation mechanisms involving vacancies or different cations.

These structural and nanoscale constraints are further reflected in the formation energetics of carbonated apatite (Fig. 4D). In contrast to pristine hydroxyapatite under the chosen chemical-potential reference states, carbonate-substituted apatite shows substantially higher formation energies. Specifically, the calculated formation energies are 127.37 kcal/mol for A-type substitution (one ${\mathrm{CO}}_{3}^{2-}$ replacing OH^−^), 204.00 kcal/mol for B-type substitution (one ${\mathrm{CO}}_{3}^{2-}$ replacing ${\mathrm{PO}}_{4}^{3-}$), and 133.10 kcal/mol for the AB-type configuration involving two carbonate substitutions. The increased formation energies strongly depend on different substitution types and vacancies or different cations for charge compensation. However, at the framework of B-type substitution using Na^+^ to balance charge, it clearly indicates that higher formation energies are derived from higher ${\mathrm{CO}}_{3}^{2-}$ concentration (Fig. 4D). These increasingly unfavorable energetics indicate that ${\mathrm{CO}}_{3}^{2-}$ incorporation thermodynamically destabilizes the apatite lattice, increasing the energetic cost of forming a well-ordered crystal. Consequently, crystal growth is inhibited and long-range order is more difficult to achieve, favoring the formation of poorly crystalline or nanoscale apatite. Such energetic constraints provide a thermodynamic basis for the structural disorder inferred from Raman band broadening, linking elevated *ν*_1_-band FWHM to carbonate-induced destabilization of the apatite lattice rather than to compositional substitution alone.

### Applications on identifying unknown apatite samples and beyond

**Figure 5:**
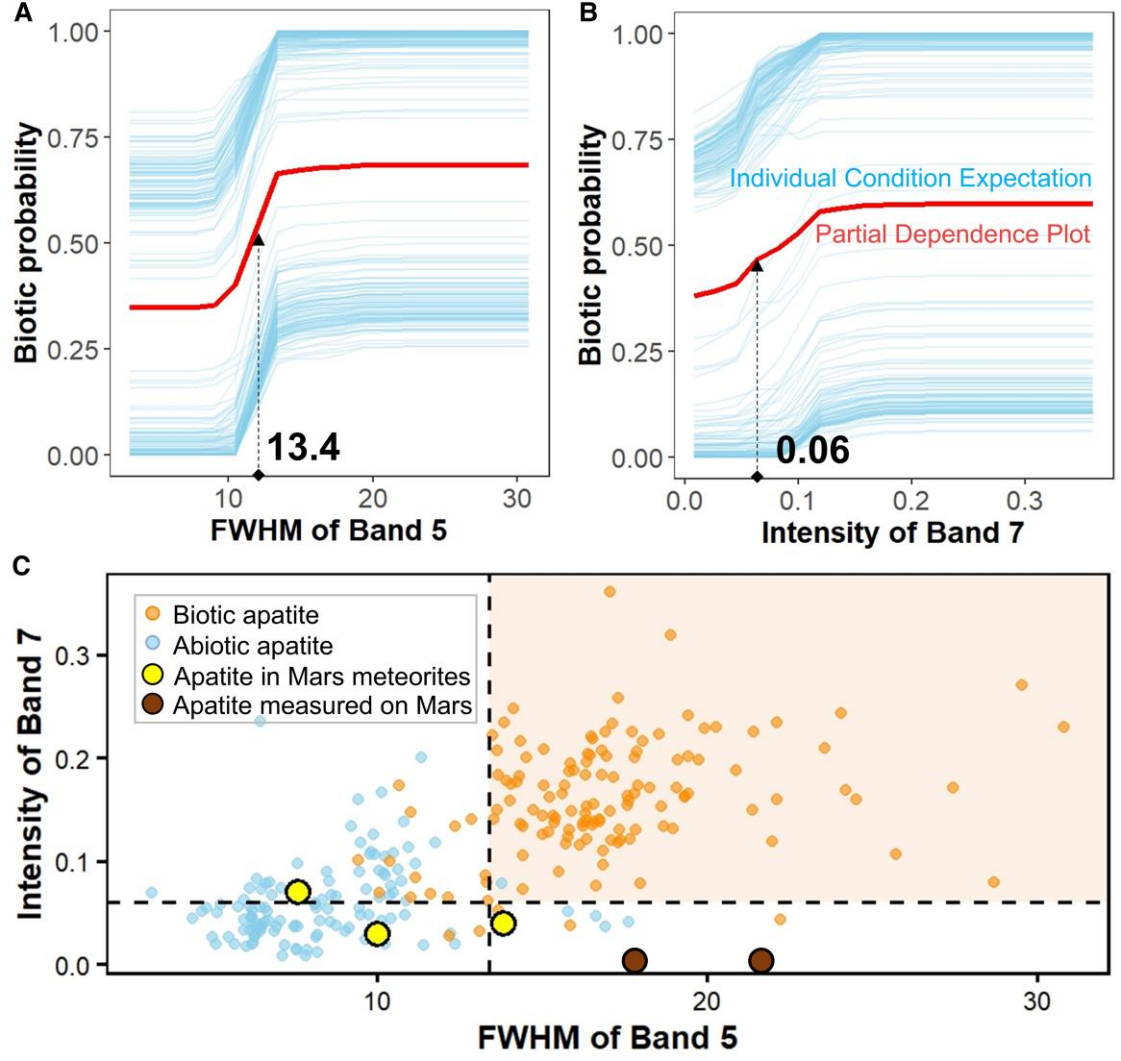
Threshold values determination of two critical Raman features and a proposed two-feature decision map for unknown apatite samples. Partial dependence (PDP) and individual conditional expectation (ICE) plots illustrating the effects of the FWHM of band 5 (*v*_1_(${\mathrm{PO}}_{4}^{3-}$)) (A) and the intensity of band 7 (overlapping *v*_3_(${\mathrm{PO}}_{4}^{3-}$) and *v*_1_(${\mathrm{CO}}_{3}^{2-}$) modes) (B) on the predicted probability of biotic apatite. Thresholds inferred from the maximum PDP slope are also shown. C) The two-feature decision map showing the high-confidence biotic region defined by values above both Raman-feature thresholds. Data points include the biotic and abiotic apatite in dataset as well as new data from three Martian meteorites and two samples measured on Mars.

To further quantify how key spectral parameters control model predictions, partial dependence and individual conditional expectation (PDP–ICE) analyses were performed for the two most influential spectral features identified by feature importance ranking (namely the FWHM of band 5 and the intensity of band 7; Fig. 2C). The PDP curves exhibit a distinct nonlinear behavior, with a pronounced increase in biotic probability over a limited range of feature values. The point of maximum slope on each PDP curve marks the region where the predicted probability is most sensitive to changes in the corresponding parameter and can therefore be used to define a characteristic threshold. Specifically, when the FWHM of band 5 exceeds ∼13.4 (Fig. 5A), the predicted probability of biotic apatite increases sharply. Similar transitions are observed for the intensity of band 7 at a value of ∼0.06 (Fig. 5B). Beyond these thresholds, the predicted biotic probability approaches a plateau, indicating diminishing marginal effects. These systematic variations in band width and intensity imply underlying structural and chemical modifications within the apatite lattice, rather than isolated spectral fluctuations.

In Fig. 5C, we present a two-feature decision map in which the FWHM of band 5 (*v*_1_(${\mathrm{PO}}_{4}^{3-}$)) and the intensity of band 7 (overlapping *v*_3_(${\mathrm{PO}}_{4}^{3-}$) and *v*_1_(${\mathrm{CO}}_{3}^{2-}$)) serve as two key diagnostic parameters to distinguish biotic apatite from abiotic counterparts. These two parameters are not only the top two critical features, but they strongly rely on prominent peak geometry rather than multipeak fitting. That means they will be minimally affected by operator-dependent peak deconvolution strategies or software-specific fitting routines. Within the four quadrants defined by these two thresholds, the first quadrant, where both FWHM of bands 5 and 7 intensity exceed their respective cutoff values (i.e. 13.4 and 0.06, respectively), is designated as a high-confidence biotic region. Projecting Raman spectra from our dataset onto this map shows that 85.8% of biotic apatite samples (115 out of 134) fall within the high-confidence region, whereas only a single abiotic apatite spectrum is misclassified into this domain. Notably, these 115 samples are consistently assigned high biotic probabilities by our supervised model, ranging from 0.859 to 1.0. This result demonstrates that the proposed two-feature decision map provides a simple yet robust framework for rapid first-order discrimination between biotic and abiotic apatite, offering an interpretable screening tool complementary to the full machine-learning classification. Three apatite crystals found in Martian meteorites (37, 38), by means of their reported Raman spectra and our model, have low probabilities to be classified as biotic (0.107, 0.082, and 0.009). When projecting them onto the two-feature decision map, as expected, they fall into the regions with low-confidence biotic interpretations. In addition, Raman spectra of apatite standards acquired by the SuperCam instrument on the Perseverance rover are consistent with those measured under laboratory conditions on Earth (19), with key spectral features showing negligible sensitivity to instrument-specific parameters. When projected into our model space (Fig. 5C), these spectra fall within the abiotic domain, providing independent support for the transferability of the framework to planetary exploration and biosignature interpretation.

## Discussion

Biological mineralization processes can introduce substantial amounts of ${\mathrm{CO}}_{3}^{2-}$ during apatite precipitation (23–27). Previous studies have primarily linked Raman band broadening in apatite to increased ${\mathrm{CO}}_{3}^{2-}$ substitution based on empirical correlations from synthetic samples. Our results extend this interpretation by showing that carbonate incorporation fundamentally destabilizes the apatite lattice, driving structural disorder and loss of crystallinity rather than acting solely as a compositional marker. Structural distortion, Raman spectral calculations, and formation-energy analyses collectively demonstrate that ${\mathrm{CO}}_{3}^{2-}$ substitution breaks the equivalence of phosphate units, inhibits long-range order, and amplifies inhomogeneous broadening of ${\mathrm{PO}}_{4}^{3-}$ bands especially the dominant *ν*_1_ band. Notably, in the apatite structure employed for calculation, Na^+^ is used to balance charge by substitute Ca^2+^ during the ${\mathrm{CO}}_{3}^{2-}$ substitution for ${\mathrm{PO}}_{4}^{3-}$ (Fig. S7). In biologically mediated apatite, however, Raman band broadening should be further enhanced by variable carbonate contents, mixed substitution types, and diverse charge-compensation mechanisms due to the apatite's structure adaptability to cation, anion, or vacancy substitutions (21, 23, 39).

An important consideration is whether abiotic apatite with elevated ${\mathrm{CO}}_{3}^{2-}$ substitution for ${\mathrm{PO}}_{4}^{3-}$ might yield a biotic false positive. Under most igneous and metamorphic environments ${\mathrm{CO}}_{3}^{2-}$ does not occur in significant solid solution with ${\mathrm{PO}}_{4}^{3-}$; however, this substitution may occur when magma or fluid is enriched in CO_2_ molecules, with C:P reaching as high as 1:4 (Fig. 1B). However, in these instances, relatively high crystallization temperatures tend to overcome the energy barriers for ${\mathrm{CO}}_{3}^{2-}$ substitution and long-range order. Within this framework, *ν*_1_-band FWHM and carbonate-related spectral features emerge as coupled, mechanistically grounded indicators of biogenesis in crystallization conditions at moderate temperatures, thus providing a physicochemical basis for the RF model used to distinguish biotic from abiotic apatite. We emphasize that ${\mathrm{CO}}_{3}^{2-}$ substitution alone is not a diagnostic indicator of the biogenic origin of apatite. Biologically mediated processes can reduce apatite crystallinity through multiple pathways (e.g. variable growth rates, incorporation of structural impurities, defect accumulation, and nanoscale domain heterogeneity), among which ${\mathrm{CO}}_{3}^{2-}$ substitution represents one important contributing factor. Moreover, we emphasize that the above conclusions are derived within the scope of the present dataset, spectral-processing workflow, and modeling framework. Future work may further expand the dataset, incorporate additional spectral descriptors (e.g. fluorescence-related baseline) and instrument-specific parameters, and explore improved modeling strategies to enhance the applicability across broader scenarios.

An important strength of our Raman-based machine-learning framework is its robustness to laboratory-specific variability in Raman data acquisition. In principle, instrumental parameters such as laser wavelength, spot size, grating configuration, and slit width can influence spectral appearance, particularly the relative intensities of certain bands. In this work, baseline correction, intensity normalization, and full-spectrum band fitting further mitigate inter-laboratory differences, allowing the extracted features to be comparable across datasets. Notably, the high performance retained under leave-one-source-out cross-validation indicates that classification is not driven by source- or instrument-specific artifacts but instead reflects intrinsic and reproducible spectral signatures distinguishing biotic and abiotic apatite. More importantly, the machine-learning framework serves to filter out extraneous experimental variability and pinpoint the key spectral parameters most relevant to biosignature discrimination. Therefore, once a Raman spectrum is acquired, normalized, and decomposed through Lorentzian fitting, the trained model can provide an objective inference of biotic or abiotic origin, together with an associated predicted probability. This procedure enables probabilistic rather than binary interpretation of apatite biosignatures, which is particularly valuable for complex or ambiguous samples encountered in planetary exploration. Alternatively, the proposed two-feature decision map provides an intuitive, transparent, and practical framework for visualizing and interpreting truly unknown samples (Fig. 5C), which remarkably facilitates practical application without the need for programming or model retraining. This model shows promise for investigating deep-time phosphatized samples (e.g. sedimentary phosphorites from the Archean and Paleozoic Eons), where biosignature interpretations are often ambiguous. Moreover, although trained on terrestrial apatite, the framework is potentially applicable to extraterrestrial settings because Raman spectral features reflect intrinsic structural disorder and chemical substitution governed by fundamental physicochemical processes, rather than environment-specific conditions. This extrapolation is also consistent with current understanding that biomineralization is controlled by common physicochemical constraints, potentially leading to comparable structural and compositional signatures in apatite across different planetary environments. Notably, with flight-ready Raman spectrometers currently operating on the Perseverance rover and planned for the Rosalind Franklin rover (17, 18), our framework offers a practical means to assess the origin of apatite identified by present and future Mars missions.

Despite the strong classification performance observed here, several important limitations and future directions remain. Firstly, the present biogenic dataset is dominated by apatite from modern terrestrial biomineralization and does not encompass the full diversity of possible phosphate-forming biological systems relevant to early Earth or extraterrestrial environments (40). Rather, we aim to identify general Raman spectral signatures associated with biologically mediated apatite formation, including increased structural disorder, carbonate incorporation, and altered crystallinity. These crystal-chemical effects may extend beyond specific biological taxa or environments. Future work incorporating microbial and environmentally mediated phosphate biominerals will help further evaluate the universality of the identified Raman biosignatures. Secondly, considering that radiation-induced structural disorder under Martian surface conditions may broaden the apatite *ν*_1_(${\mathrm{PO}}_{4}^{3-}$) Raman band (19), which is consistent with our observations of disorder-related phosphate-band broadening, irradiated carbonate-bearing abiotic apatite could potentially increase spectral similarity to some biogenic populations. Future studies incorporating irradiated and carbonate-bearing abiotic apatite will help further evaluate potential applications under extraterrestrial conditions. Thirdly, biogenic apatite is commonly associated with nanoscale particle organization and organic matrix templates such as collagen and related macromolecular scaffolds (20), which may influence Raman spectral properties beyond crystal-chemical effects alone. Although such organic components may be less stable than lattice-bound carbonate over geological timescales, especially in ancient or extraterrestrial samples, their potential spectral influence and preservation deserve further investigation.

More broadly, our results highlight the growing importance of combining flight-ready analytical instruments with AI to fully exploit the information content of in situ planetary datasets. Rather than relying on single spectral features or qualitative assessment, data-driven approaches enable multivariate, probabilistic interpretations under realistic mission constraints (5, 28, 41). Crucially, the interpretability of the identified features, linking Raman band shifting, broadening, and intensity variations to crystal-chemical and energetic mechanisms, ensures that machine-learning predictions remain physically grounded rather than purely empirical (42, 43).

As planetary exploration increasingly shifts toward autonomous decision-making and target selection (44, 45), the integration of interpretable AI with mineralogical biosignatures offers a powerful and extensible framework. Beyond minerals alone, future life-detection strategies will increasingly rely on the joint interpretation of mineralogical and organic spectroscopic signatures derived from on-board py-GC-MS, infrared spectroscopy, luminescence, laser-induced breakdown spectroscopy, X-ray fluorescence, and other analytic methods. During data decoding, interpretable AI enables the systematic fusion of these heterogeneous datasets (46, 47), allowing biologically mediated patterns to be distinguished from abiotic backgrounds in a physically and chemically consistent manner. In this context, such an integrative and interpretable framework provides a pathway toward autonomous, mechanism-informed life detection across diverse planetary environments, advancing the broader goals of planetary science in understanding habitability, biosignature preservation, and the coevolution of geology and biology.

## Materials and methods

### Spectral data collection and processing

The 331 Raman spectra used in this study are from peer-review publications (182 spectra) (25, 27, 33, 48–114), laboratory measurements (121 spectra), and public databases (28 spectra). For Raman spectra available only as graphical representations in public databases or peer-reviewed publications, spectral curves were digitized using GetData Graph Digitizer (version 2.22). The majority of extracted spectra had sampling intervals within 2 cm^−1^, with substantially finer resolution (typically ∼0.5 cm^−1^ or less) around peak positions. For spectra lacking numerical intensity scales (e.g. reported in arbitrary units), an arbitrary intensity range was assigned during digitization. The absolute intensity scale was not used in subsequent analyses, as all spectra were subsequently normalized to their strongest peak and rescaled to values between 0 and 1. Public databases include RRUFF (https://www.rruff.net/; 21 spectra), a dataset hosted by the École Normale Supérieure de Lyon (https://lithotheque.ens-lyon.fr/Raman/raman.php; four spectra), and HORIBA dataset from commercial software (LabRAM, HORIBA, Japan; three spectra), all of which provide a total of 21 raw data and seven graphical spectral curves.

For laboratory measurements of apatite, we have four kinds of samples: gem-quality fluorapatite crystal samples from Madagascar, Africa (24 spectra), carbonate-fluorapatite samples in phosphorite rocks from the Weng’an deposit, China (31 spectra), modern pathological calcification mainly as hydroxyapatite and carbonate-hydroxylapatite (51 spectra), and synthetic carbonate-hydroxylapatite (15 spectra). For the Madagascar apatite, Raman spectroscopy was performed with a Raman spectrometer (LabRAM, HORIBA) with a 532-nm laser wavelength (114), while the spectra of the rest of the samples were collected on the Raman spectrometer (inVia Reflex, Renishaw, United Kingdom) under a 785-nm line. For sedimentary phosphorites (115), thin sections of rock samples were used. For human pathological calcification samples, specimens were derived from ligamentum flavum (32 spectra), atherosclerotic plaques (nine spectra), breast tumors (eight spectra), and thyroid tissue (two spectra). Paraffin-embedded tissue blocks were obtained from the Department of Pathology, Peking University Health Science Center, in accordance with institutional ethical guidelines and informed consent procedures. Following standard histopathological protocols (116, 117), thin sections ∼10 μm in thickness and 15 mm × 15 mm in area were prepared from the paraffin-embedded blocks using a Leica ultramicrotome. The sections were mounted onto silicon wafers and subsequently subjected to successive xylene and ethanol treatments for deparaffinization.

Spectral preprocessing and peak fitting on all 311 spectra were conducted using OriginPro 2022 (version 9.9.0.225). Raw Raman spectra were baseline corrected to remove background fluorescence and instrumental offsets, followed by normalization to the intensity of the strongest peak in each spectrum to facilitate inter-sample comparison. Full-spectrum peak deconvolution was performed using Lorentzian line shapes, which are commonly employed to represent vibrational Raman modes (118, 119). In the case of apatite, we find that Lorentzian line shapes provide consistently better fits than Gaussian functions, while more complex Voigt-type profiles (mixed Lorentzian–Gaussian functions) do not yield appreciable improvements in fitting quality. Peak positions, widths, and intensities were simultaneously optimized during fitting. In total, 331 spectra were analyzed. The resulting goodness-of-fit values (*R*^2^) ranged from 0.8646 to 0.9777, with an average value of 0.9730, demonstrating robust and consistent fitting quality across the entire dataset.

### RF classification

An RF classifier was developed to discriminate abiotic and biotic apatite using a 21-dimensional set of Raman-derived spectral features. The full dataset comprised 255 spectra, including 121 abiotic and 134 biotic spectra, but excluding 76 synthetic ones. The data were randomly divided into a training set (75%) and an independent hold-out test set (25%) using stratified sampling to preserve class proportions. The test set was not used at any stage of model training or hyperparameter tuning. Binary classification was performed, with biotic apatite samples defined as the positive class.

Model training and hyperparameter optimization were performed exclusively on the training set using repeated stratified 10-fold cross-validation (10 repeats). RF models were implemented using the ranger engine (version 0.17.0) within the caret framework (version 7.0.1) in R (version 4.5.2). Candidate hyperparameters included the number of variables randomly sampled at each split (*mtry*), the minimum terminal node size (*min.node.size*), and the split rule. Model performance during cross-validation was evaluated using the AUC, computed from class probabilities, and the optimal hyperparameter combination was selected by maximizing cross-validated AUC.

The final model employed *mtry* = 5, *min.node.size* = 3, the *extratrees* split rule, and 500 trees. The number of trees was determined based on convergence of the out-of-bag error, with 500 trees representing the smallest value at which model error stabilized. Following hyperparameter selection, the RF model was refit on the full training dataset using these optimal settings. Model performance was assessed on the independent test set using AUC together with threshold-dependent metrics derived from the confusion matrix, including sensitivity, specificity, F1 score, and Matthews correlation coefficient. Class labels were assigned using a probability threshold of 0.5 for the positive class (biotic apatite). Feature contributions were evaluated using permutation importance as implemented in ranger.

To further examine model robustness and to assess potential information leakage, additional validation schemes were conducted on the training set using the optimized hyperparameters. Leave-one-out cross-validation was performed by iteratively withholding a single spectrum from the training set and predicting its class using a model trained on all remaining spectra, from which cross-validated accuracy and AUC were computed. In addition, because the full dataset comprises 255 spectra collected from 60 distinct sources (54 peer-reviewed publications, three public databases, and three in-house measurement sets representing three sample groups acquired on three instruments), spectra originating from the same source may share acquisition conditions and sample-specific characteristics that could inflate performance if split across training and testing subsets. We therefore applied leave-one-source-out cross-validation, in which all spectra from one source were withheld as a group while the model was trained on the remaining sources; pooled out-of-source predictions were then used to calculate overall training-set accuracy and AUC. As a negative control, label permutation was conducted by randomly shuffling the abiotic/biotic class assignments within the training set prior to cross-validation. The accuracies and AUC values from complementary validation procedures are summarized in Table 2.

All analysis scripts are publicly available on GitHub. The full dataset used in this study is provided both as a supplementary database accompanying the article and in the same GitHub repository.

### Raman spectra and formation-energy calculations

Raman spectral calculations and formation-energy analyses were conducted to quantify the effects of carbonate substitution on the vibrational properties and thermodynamic stability of apatite. All calculations were based on a hydroxyapatite unit cell containing six ${\mathrm{PO}}_{4}^{3-}$ groups as the pristine reference structure, except for the C:P = 1:11 substitution case, which was modeled using a 2 × 1 × 1 hydroxyapatite supercell. Carbonate substitution was introduced by replacing a ${\mathrm{PO}}_{4}^{3-}$ group, an OH^−^ group, or both ${\mathrm{PO}}_{4}^{3-}$ and OH^−^ sites with ${\mathrm{CO}}_{3}^{2-}$, corresponding to B-, A-, and AB-type substitutions, respectively. These configurations yield C-to-P ratios of 1:11, 1:5, and 1:2 for B-type substitution, 1:6 for A-type substitution, and 2:5 for AB-type substitution (Fig. S7 and Table S4). Charge compensation associated with ${\mathrm{CO}}_{3}^{2-}$ incorporation was achieved by Na^+^ substitution for Ca^2+^ where required (120, 121).

Density-functional theory (DFT) calculations were performed using the projected augmented wave (PAW) method as implemented in the Vienna *Ab initio* Simulation Package (122–126). The exchange-correlation effects were described within the generalized gradient approximation using the Perdew–Burke–Ernzerhof functional (127). The ion–electron interactions were treated using PAW pseudopotentials. The plane wave energy cutoff was set to 700 eV and the energy convergence criterion for the electronic self-consistent calculations was 10^−6^ eV. The force difference was converged to 1 × 10^−3^ eV/Å. The *K*-point grids were set as 3 × 3 × 2 in the Brillouin zone for apatite in structural relaxation calculations using the Monkhorst–Pack method (128). All structures were fully relaxed to find configurations with minimal interatomic forces and hydrostatic residual stresses (Fig. S7). Then, we employed the density-functional perturbation theory (129, 130) to compute the dynamical matrices as energy derivatives with respect to atomic displacements and electric fields. The diagonalization of the dynamical matrices yields the vibrational frequencies and the corresponding atomic displacement patterns. These results were then used to compute the force-constant Hessian matrix and the elementary second derivative response-function tensors (131).

Formation-energy calculations were conducted to assess the relative thermodynamic stability of carbonate-substituted hydroxyapatite with respect to pristine hydroxyapatite. For each substitution configuration (A, B, and AB types), the formation energy (Δ*E*_f_) was evaluated using a reaction-based formalism (Eq. 1):


(1) $$\Delta E_{f}=E_{\mathrm{tot}}(\mathrm{Substituted}\;\mathrm{HAP})-E_{\mathrm{tot}}(\mathrm{HAP})-\sum_{i}\Delta n_{i}\mu_{i},$$

where *E*_tot_ denotes the fully relaxed total energy, Δ*n_i_* represents the change in the number of atoms of species *i* between the substituted and pristine structures (positive for addition and negative for removal), and *μ_i_* is the chemical potential of element *i* referenced to its stable elemental phase on a per-atom basis (Table S5).

As an illustrative example, for A-type substitution with a C-to-P ratio of 1:6 (Table S4), comparison between the substituted structure and pristine hydroxyapatite shows that the net compositional changes involve C, O, and H, with Δ*n*_C_ = +1, Δ*n*_O_ = +1, and Δ*n*_H_ = −2, respectively. This formulation explicitly accounts for the removal and incorporation of the corresponding ionic species as well as charge-compensating substitutions required by carbonate incorporation.

By using a consistent set of chemical potentials for all configurations, the calculated formation energies provide a robust basis for comparing energetic trends among different substitution modes, while minimizing sensitivity to the absolute choice of chemical-potential reference. Detailed numerical results and reference energies are listed in Table S6.

### Distortion quantification of phosphate tetrahedrons

Local structural distortion induced by ${\mathrm{CO}}_{3}^{2-}$ substitution was quantified by the bond-length distortion index and the tetrahedral angle variance of ${\mathrm{PO}}_{4}^{3-}$ units (34, 35). Optimized configurations of pristine hydroxyapatite, A-, B-, and AB-type substitutions were derived from DFT calculations. Values of P–O bond length and O–P–O bond angle were calculated by R package Crystract based on Crystallographic Information Files (Table S2 and S3).

## Supplementary Material

pgag215_Supplementary_Data

## Data Availability

The data generated and analyzed in this manuscript can be found on the Open Science Framework repository titled “Apatite biosignature detection based on Raman spectroscopy and machine learning” (https://osf.io/njz6c/overview). The code for the paper can be found at https://github.com/YanzhangLi-AI/Apatite-Raman-ML. All data, code, and materials used in the analysis are available to any researcher for purposes of reproducing or extending the analysis. Licenses for the data and code usage and relevant attribution information will be updated on the respective repositories.
