# Supplementary material for: Mineral biosignature identification from Raman spectroscopy using machine learning
Source: PNAS Nexus. 2026 Jul 14;5(7):pgag215. doi: 10.1093/pnasnexus/pgag215 (PMC13366543; doi:10.1093/pnasnexus/pgag215)
Supplement: pgag215_Supplementary_Data [file pgag215_supplementary_data.docx]

**
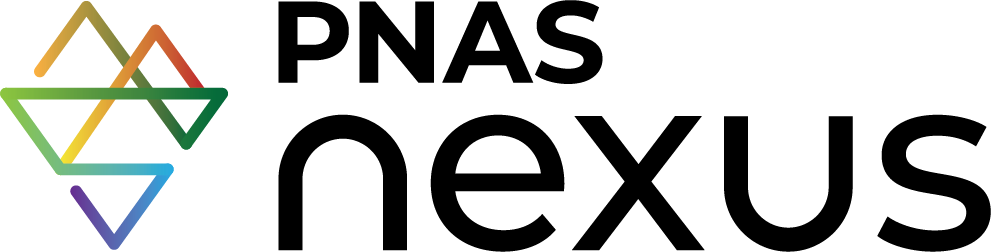
**

**Supplementary Information for**

Mineral biosignature identification from Raman spectroscopy using machine learning.

Yanzhang Li, Anirudh Prabhu, Bingxu Hou, Michael L. Wong, Anhuai Lu, Jieqi Xing, Don Ngo, Bo Xu, Robert M. Hazen

Corresponding author: Robert M. Hazen

**Email:**  rhazen@carnegiescience.edu

**This PDF file includes:**

Figures S1 to S7

Tables S1 to S6


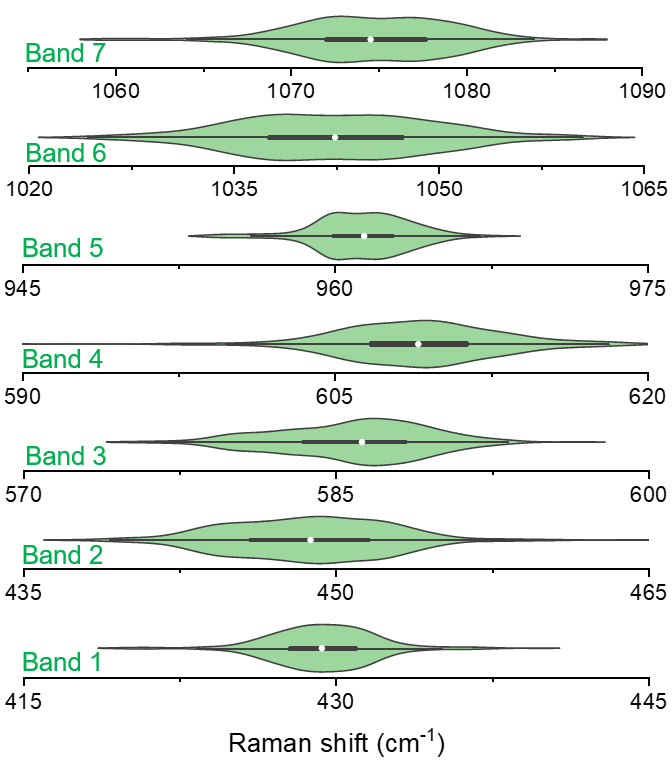


Figure S1. Violin plots of fitted Raman peak positions for seven bands, demonstrating the consistency of band positions across 331 Raman spectra. Each violin plot shows a miniature box-and-whisker plot giving the median (white dot), upper and lower quartiles (box), and extent of distribution (whiskers).


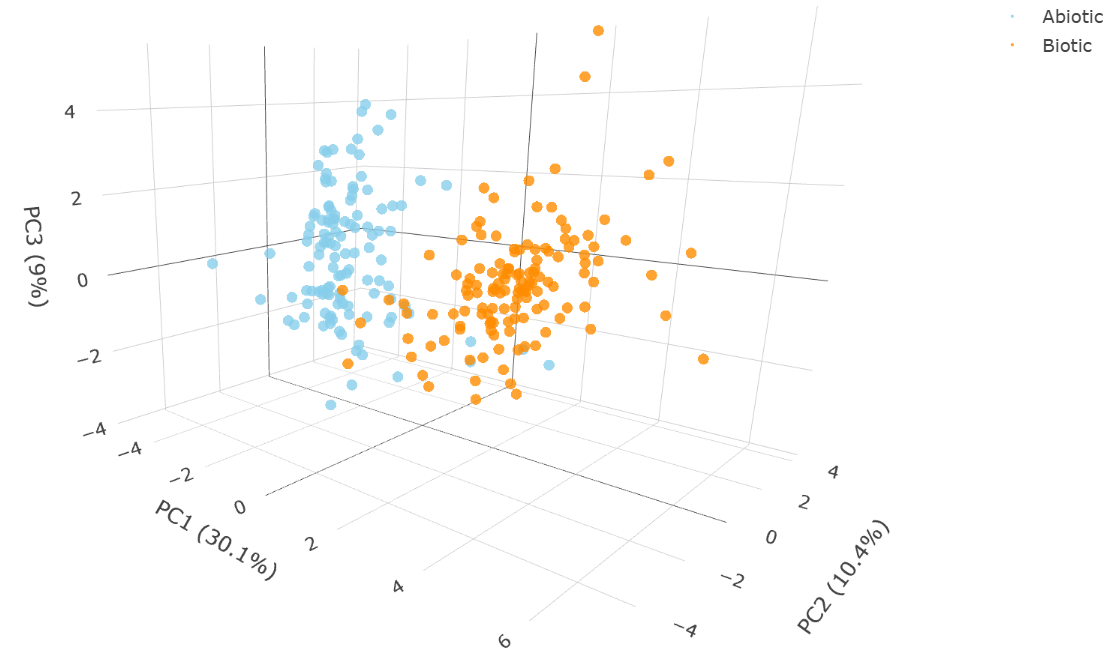


Figure S2. Three-dimensional principal component analysis (PCA) of abiotic and biotic apatite samples based on 21-dimensional Raman-derived spectral features. Scores on the first three principal components (PC1, PC2, and PC3) are shown, accounting for a combined 49.5% of the total variance. Points represent individual samples coloured by endmember classification.


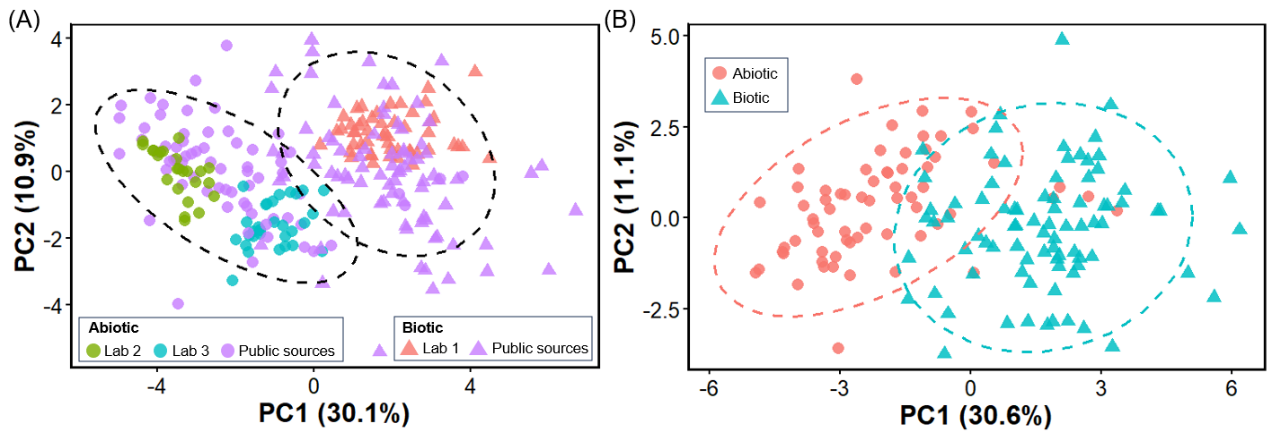


Figure S3. Principal component analysis (PCA) of apatite Raman spectra based on 21 Raman-derived spectral features. (A) PCA colored by analytical source and biological origin. Internal laboratory datasets (Lab 1–3) and literature/public datasets are shown together to evaluate the influence of source-related analytical variability on spectral clustering patterns. (B) PCA of literature/public datasets only after exclusion of all internally acquired spectra, showing that separation between biogenic and abiotic apatite remains preserved independently of internal laboratory datasets.


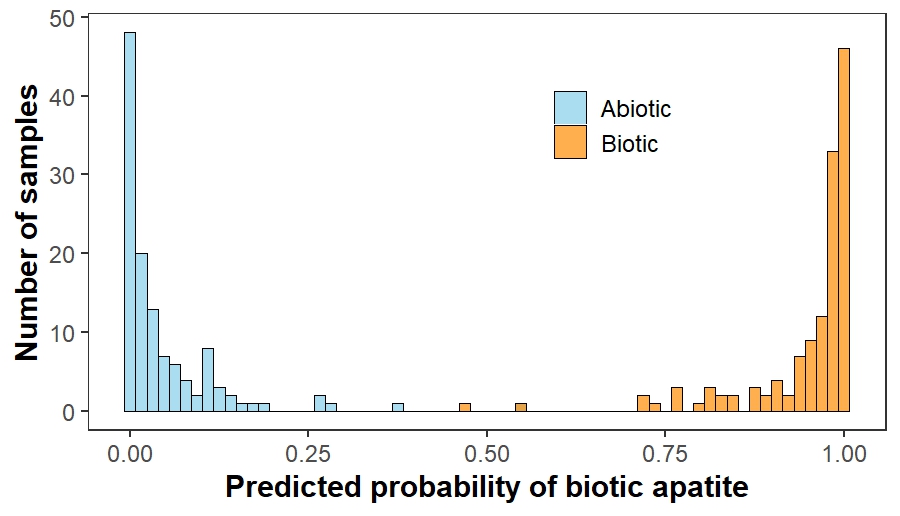


Figure S4. Distribution of predicted biogenesis probabilities for abiotic and biogenic apatite samples based on the trained random forest model. Colors correspond to endmember classification.


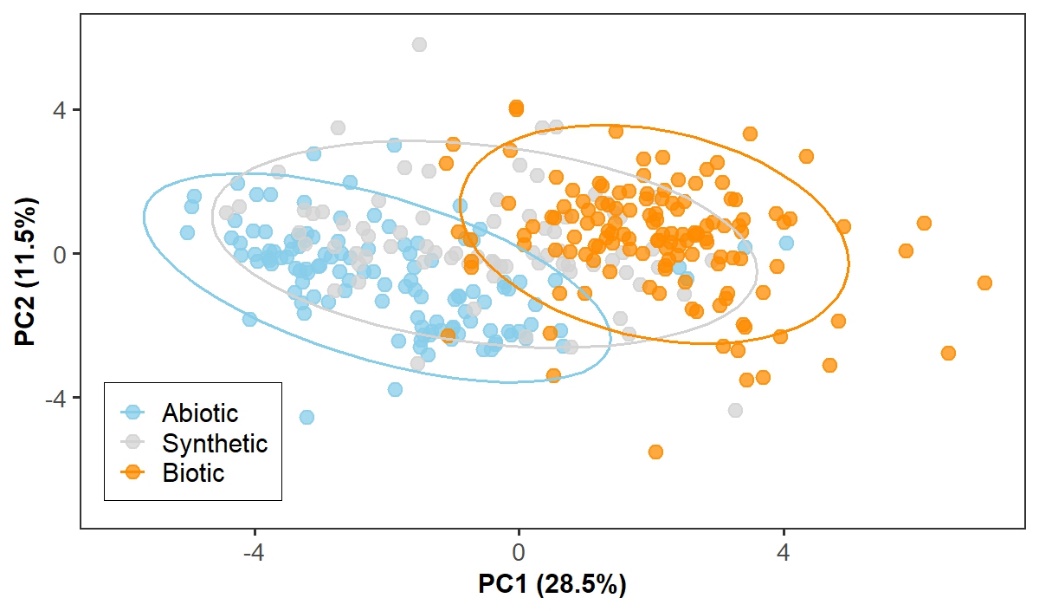


Figure S5. Principal component analysis (PCA) of abiotic, biotic, and synthetic apatite samples based on 21-dimension Raman-derived spectral features. Scores on the first two principal components (PC1 and PC2) are shown, accounting for a combined 40.0% of the total variance. Points represent individual samples coloured by endmember classification, and ellipses indicate the 95% data dispersion for each class assuming a multivariate *t* distribution.


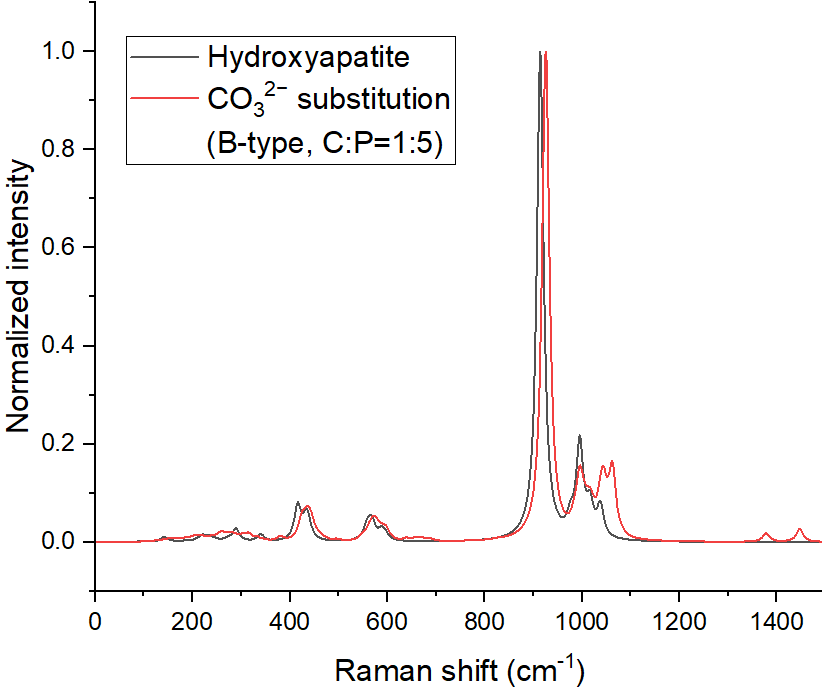


Figure S6. Calculated Raman spectra of pristine hydroxyapatite and carbonated hydroxyapatite with CO_3_^2−^ substituting for PO_4_^3−^ in the C-to-P ratio of 1:5.


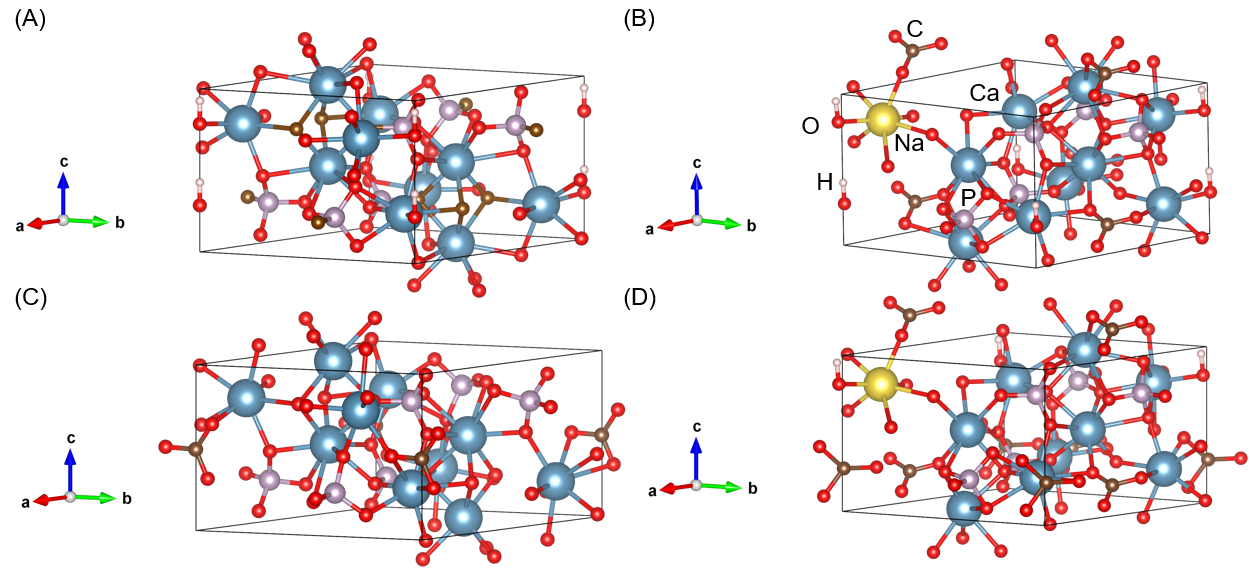


Figure S7. The optimal structures for Raman spectra calculations. (A) Pristine hydroxyapatite. (B) Hydroxyapatite with CO_3_^2−^ substituting for PO_4_^3−^ in the C-to-P ratio of 1:5 (B-type). (C) Hydroxyapatite with CO_3_^2−^ substituting for OH^−^ in the C-to-P ratio of 1:6 (A-type). (D) Hydroxyapatite with CO_3_^2−^ substituting for both PO_4_^3−^ and OH^−^ in the C-to-P ratio of 2:5 (AB-type).

Table S1. Cross-validated performance of different random forest classifier metrics. Values are reported as mean ± standard deviation (SD) over repeated 10-fold cross-validation (10 repeats) in training test. The standard deviation of MCC could not be reliably estimated due to undefined values in some cross-validation folds.

| Metric | Mean | SD |
| --- | --- | --- |
| AUC | 0.998 | 0.0055 |
| Sensitivity | 0.982 | 0.0478 |
| Specificity | 0.956 | 0.0581 |
| F1 score | 0.968 | 0.0370 |
| MCC | 0.942 | NA |

Table S2. P-O bond lengths in phosphate tetrahedra in four optimal structures of hydroxyapatite.

| **Structure** | **PO_4_^3−^ tetrahedrons**  (6 or 5) | **P−O Bonds** (4 in total) (Å) | | | |
| --- | --- | --- | --- | --- | --- |
|  |  | #1 | #2 | #3 | #4 |
| Pristine hydroxyapatite | 6 equivalents | 1.5508 | 1.5580 | 1.5486 | 1.5471 |
| One CO_3_^2-^ substitutes for one PO_4_^3−^  (B-type) | #1 | 1.5425 | 1.5435 | 1.5552 | 1.5593 |
|  | #2 | 1.5611 | 1.5446 | 1.5570 | 1.5489 |
|  | #3 | 1.5669 | 1.5586 | 1.5475 | 1.5398 |
|  | #4 | 1.5372 | 1.5564 | 1.5469 | 1.5632 |
|  | #5 | 1.5433 | 1.5561 | 1.5451 | 1.5552 |
| One CO_3_^2-^ substitutes for one OH^−^  (A-type) | #1 | 1.5562 | 1.5496 | 1.5474 | 1.5658 |
|  | #2 | 1.5465 | 1.5514 | 1.5556 | 1.5607 |
|  | #3 | 1.5411 | 1.5581 | 1.5555 | 1.5641 |
|  | #4 | 1.5572 | 1.5599 | 1.5357 | 1.5559 |
|  | #5 | 1.5664 | 1.5489 | 1.5530 | 1.5526 |
|  | #6 | 1.5656 | 1.5391 | 1.5520 | 1.5578 |
| Two CO_3_^2-^ substitute for one PO_4_^3−^ and one OH^−^  (AB-type) | #1 | 1.5425 | 1.5552 | 1.5436 | 1.5593 |
|  | #2 | 1.5489 | 1.5612 | 1.5446 | 1.5570 |
|  | #3 | 1.5670 | 1.5586 | 1.5474 | 1.5399 |
|  | #4 | 1.5373 | 1.5564 | 1.5469 | 1.5632 |
|  | #5 | 1.5434 | 1.5561 | 1.5451 | 1.5552 |

Table S3. P-O-P bond angles in phosphate tetrahedra in four optimal structures of hydroxyapatite.

| **Structure** | **PO_4_^3−^ tetrahedra**  (6 or 5) | **O−P−O Angles** (6 in total) (º) | | | | | |
| --- | --- | --- | --- | --- | --- | --- | --- |
|  |  | #1 | #2 | #3 | #4 | #5 | #6 |
| Pristine hydroxyapatite | 6 equivalents | 111.15 | 111.02 | 111.95 | 108.10 | 107.02 | 107.40 |
| One CO_3_^2-^ substitutes for one PO_4_^3−^  (B-type) | #1 | 106.59 | 109.30 | 110.66 | 112.85 | 111.92 | 105.22 |
|  | #2 | 106.90 | 110.67 | 108.15 | 110.12 | 109.65 | 111.23 |
|  | #3 | 106.22 | 107.52 | 112.59 | 107.16 | 110.73 | 112.27 |
|  | #4 | 114.11 | 110.02 | 111.53 | 105.35 | 106.18 | 109.37 |
|  | #5 | 107.27 | 110.25 | 110.43 | 107.00 | 110.24 | 111.51 |
| One CO_3_^2-^ substitutes for one OH^−^  (A-type) | #1 | 107.22 | 106.76 | 112.04 | 112.62 | 110.24 | 107.68 |
|  | #2 | 106.82 | 113.92 | 106.22 | 110.59 | 108.80 | 110.26 |
|  | #3 | 107.47 | 109.61 | 108.99 | 112.12 | 105.58 | 112.85 |
|  | #4 | 110.08 | 103.84 | 110.34 | 112.73 | 107.52 | 112.33 |
|  | #5 | 104.97 | 107.47 | 112.01 | 108.64 | 112.61 | 110.84 |
|  | #6 | 104.57 | 111.63 | 110.32 | 111.08 | 112.64 | 106.70 |
| Two CO_3_^2-^ substitute for one PO_4_^3−^ and one OH^−^  (AB-type) | #1 | 106.60 | 110.65 | 109.31 | 112.85 | 105.22 | 111.92 |
|  | #2 | 108.15 | 109.65 | 111.22 | 106.90 | 110.67 | 110.12 |
|  | #3 | 106.22 | 107.52 | 112.59 | 107.16 | 110.73 | 112.27 |
|  | #4 | 114.11 | 110.02 | 111.53 | 105.35 | 106.18 | 109.36 |
|  | #5 | 107.27 | 110.25 | 110.43 | 107.00 | 110.24 | 111.51 |

Table S4. Substitution types, C-to-P ratios, and corresponding formulas in density functional theory (DFT) calculations.

| Substitutions | C-to-P  ratios | Substitution formulas |
| --- | --- | --- |
| B-type | 1:11 |  |
|  | 1:5 |  |
|  | 1:2 | $2Ca_{5}(PO_{4})_{3}(OH)+2CO_{3}^{2-}+2Na^{+}\to2Ca_{4}Na(PO_{4})_{2}(CO_{3})(OH)+2PO_{4}^{3-}+2Ca^{2+}$ |
| A-type | 1:6 | $2Ca_{5}(PO_{4})_{3}(OH)+CO_{3}^{2-}\to2Ca_{5}(PO_{4})_{3}(CO_{3})_{0.5}+2OH^{-}$ |
| AB-type | 2:5 |  |

Table S5. Calculated chemical potential of elements.

| Elements | Stable elemental reference phases | Total energy (eV) | The number of atoms in reference cell | Chemical potential of elements (eV/atom) |
| --- | --- | --- | --- | --- |
| C | Graphite | −36.910 | 4 | −9.228 |
| Ca | Calcium metal | −7.692 | 4 | −1.923 |
| H | H_2_ molecule | −0.556 | 2 | −0.278 |
| Na | Sodium metal | −2.596 | 2 | −1.298 |
| O | O_2_ molecule | −77.211 | 16 | −4.826 |
| P | Black phosphorus | −42.814 | 8 | −5.352 |

Table S6. Calculated formation energies (Δ*E_f_*) of different CO_3_^2−^-substituted hydroxyapatite (HAP) species compared to the ideal end-member (with total energy −311.079 eV).

| Substitution type | C-to-P ratios | Total energy (eV) | Change in the number of atoms and corresponding elements | Δ*E_f_*  (eV) | Δ*E_f_*  (kcal/mol) |
| --- | --- | --- | --- | --- | --- |
| B-type | 1:11^1^ | −612.140 | +1 (C), +1 (Na), −1 (O), −1 (P), −1 (Ca) | 8.442 | 194.682 |
|  | 1:5 | −300.658 | +1 (C), +1 (Na), −1 (O), −1 (P), −1 (Ca) | 8.846 | 203.996 |
|  | 1:2 | −290.753 | +2 (C), +2 (Na), −2 (O), −2 (P), −2 (Ca) | 17.176 | 396.075 |
| A-type | 1:6 | −319.052 | +1 (C), +1 (O), −2 (H) | 5.523 | 127.370 |
| AB-type | 2:5 | −322.958 | +2 (C), +1 (O), −1 (H), −1 (P) | 5.772 | 133.100 |

Note: ^1^This example incorporates the change between the substituted HAP and two HAP unit cells.
